# Variation in Prevertebral Soft Tissue Swelling after Staged Combined Multilevel Anterior–Posterior Complex Cervical Spine Surgery: Anterior Then Posterior (AP) versus Posterior Then Anterior–Posterior (PAP) Surgery

**DOI:** 10.3390/jcm11237250

**Published:** 2022-12-06

**Authors:** Dong-Chan Eun, Anthony A. Suguitan, Kyung-Soo Suk, Hak-Sun Kim, Ji-Won Kwon, Seong-Hwan Moon, Yong-Ho Lee, Byung Ho Lee

**Affiliations:** Department of Orthopedic Surgery, College of Medicine, Yonsei University, Seoul 03722, Republic of Korea

**Keywords:** cervical, prevertebral soft tissue swelling, anterior–posterior cervical spine surgery, complex cervical spine surgery

## Abstract

The influence of the sequence of surgery in the development of prevertebral soft tissue swelling (PSTS) in staged combined multilevel anterior–posterior complex spine surgery was examined. This study was conducted as a retrospective study of patients who underwent staged combined multilevel anterior–posterior complex cervical spine surgery from March 2014 to February 2021. Eighty-two patients were identified, of which fifty-seven were included in the final analysis after screening. PSTS was measured from routine serial monitoring lateral cervical radiographs prior to and after surgery for five consecutive days at each cervical level from C2 to C7 in patients who underwent anterior then posterior (AP) and posterior then anterior–posterior (PAP) surgery. The mean PSTS measurements significantly differed from the preoperative to postoperative monitoring days at all cervical levels (*p* = 0.0000) using repeated measures analysis of variance in both groups. PSTS was significantly greater in PAP than in AP at level C2 on postoperative day (POD) 1 (*p* = 0.0001). PSTS was more prominent at levels C2–4 during PODs 2–4 for both groups. In staged combined multilevel anterior–posterior complex spine surgery, PSTS is an inevitable complication. Therefore, surgeons should monitor PSTS after surgery when performing anterior–posterior complex cervical spine surgery, especially in the immediate postoperative period after PAP surgery.

## 1. Introduction

Combined anterior–posterior surgery is usually performed for complex cervical spine cases with multilevel pathologies that require both anterior and posterior decompression, correction of kyphosis, restoration of instability, and attainment of sagittal balance [1,2,3,4]. Staging the procedures with sufficient interval days in between the surgeries deters the deleterious effects of prolonged surgery and anesthesia time [3].

Prevertebral soft tissue swelling (PSTS) has been shown to cause postoperative problems, such as airway compromise and dysphagia, in anterior cervical spine surgery [2,3,5,6,7]. In nonstaged combined anterior–posterior cervical spine surgery, researchers have indicated that the dependent position while the patient is prone during the posterior surgery contributes to the development of PSTS and laryngopharyngeal edema postoperatively [7,8]. Our institution has experience in treating complex multi-level degenerative and deformity cervical spine cases in a staged combined anterior–posterior fashion with two different set sequences for the anterior and posterior surgeries: the two sequences include anterior first then posterior (AP) and posterior first then both anterior and posterior in the second surgery (PAP). The objective of this work was to investigate if there is a difference in PSTS between AP and PAP staged combined multilevel anterior–posterior complex cervical spine surgery at different cervical levels across different postoperative days after surgery.

## 2. Materials and Methods

This study was conducted after obtaining institutional review board approval from our institution (IRB number 4-2019-1279). The records of patients from March 2014 to February 2021 were retrospectively reviewed. Subjects that were included comprised patients who underwent combined treatment for multilevel complex degenerative and deformity cervical spine conditions. We excluded patients who had cervical spine pathologies involving trauma, tumor, or infection. We also excluded patients who underwent anterior cervical corpectomy fusion for any cervical spine condition. In addition, only patients who underwent anterior cervical discectomy fusion (ACDF) of at least three levels from C2 to T1 were selected. We collected data on the demographic and surgical profiles of the patients.

### 2.1. PSTS Measurement and Subject Cohort

**Figure 1:**
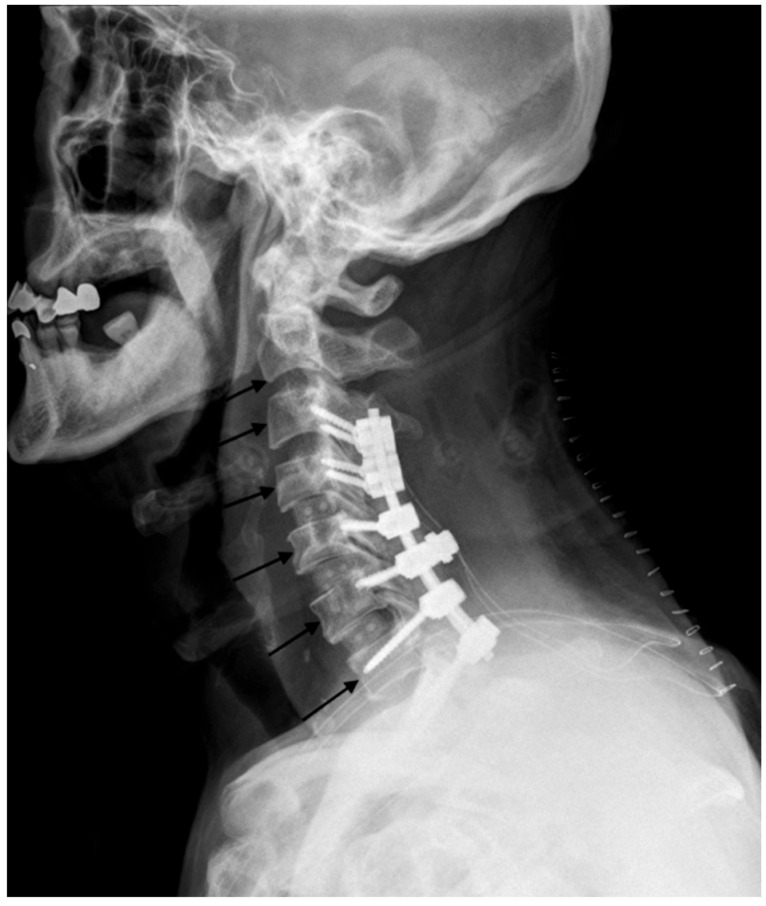
Plain radiograph showing PSTS measurements.

Preoperative and routine serial postoperative lateral monitoring cervical spine radiographs from postoperative days (POD) 1 to 5 were reviewed [9,10,11]. The postoperative X-ray was taken after the second operation. PSTS was measured as the distance in millimeters along a line from the mid anterior vertebral body surface, parallel to the upper end plate, up to the airway shadow (Figure 1) from C3 to C7 using Centricity TM Web PACS Viewer software (General Electric, Milwaukee, WI, USA). At level C2, PSTS was measured from the posteroinferior body surface of the vertebral body surface parallel to the lower C2 endplate. Two independent observers (two spine fellows) measured the values, and each observer repeated the measurements with 2-week intervals. To improve the accuracy before the measurement, we measured the values for five patients who were not subjects as measurements in advance. The magnification ratio used for the rehearsal measurement was the same for the real measurement.

**Figure 2:**
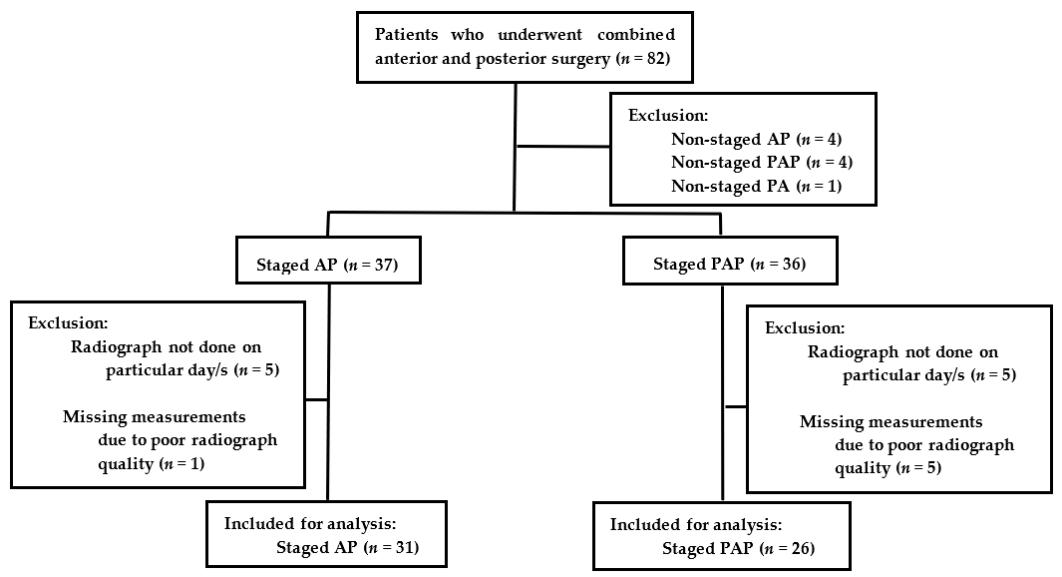
Flow diagram of the subject cohort.

Eighty-two patients were initially included, and nine were excluded for being nonstaged. Of the staged AP patients (*n* = 37), six were excluded for either radiographs not having been acquired on particular days during the monitoring or missing measurements due to poor radiograph quality. Among the staged AP group, only 31 were included for the final analysis. Of the staged PAP patients (*n* = 36), 10 were excluded for the reasons above. Among the staged PAP group, only 26 were included for the final analysis (Figure 2).

### 2.2. Surgical Procedure

Standard ACDF was performed for anterior surgery utilizing a left-sided Smith-Robinson approach. Adjacent vertebral body Caspar pin distraction was performed to the greatest extent possible. Disc space and endplate preparation were carried out by curettage and the use of burrs. Decompression procedures, such as anterior foraminotomy or en bloc uncinate resection, were also employed. The interbody fusion spacers used included composite lordotic cortical–cancellous allograft blocks (Cornerstone-ASR, Medtronic Sofamor Danek, Memphis, TN, USA) and bioactive glass–ceramic spacers (NovoMaxTM, BioAlpha Inc., Seong-nam, Republic of Korea). No anterior instrumentation or plate was used to avoid limiting any posterior correction of kyphosis that could be achieved with posterior surgery, except in some cases when a plate (VENTURETM Anterior Cervical Plate System, Medtronic Sofamor Danek, Memphis, TN, USA) was used according to the surgeons’ discretion at levels C7-T1 to prevent subsidence.

Posterior surgery was accomplished using a posterior approach to the cervical spine. Decompression of the cord was primarily attained by multi-segmental en bloc laminectomy. Additional decompression methods were also employed, such as posterior foraminotomy, laminotomy, and dome laminoplasty. A combination of a lateral mass and pedicle screw SYNAPSETM system (DePuySynthes, Mississauga, ON, Canada) or an all-pedicle screw POSEIDONTM system (Medyssey, Seoul, Republic of Korea) with a rod construct was used for fixation. A rod connector was used as necessary to properly link the rod with the screws in the lateral mass and pedicle screw combination construct. A crosslink was usually placed at the middle of the construct for added stability. Residual kyphosis correction was established by segmental instrumentation, which also allowed for a sustained alignment and promoted fusion. The posterior fusion and decompression levels were selected on the basis of levels of compression and the preservation of sagittal balance [12]. Autologous bone grafts were used for the fusion. No intraoperative local steroid or recombinant human bone morphogenetic protein 2 was administered in any surgeries [13].

In AP surgery, ACDF was performed in the first surgery stage, followed by posterior decompression and fusion in the second surgery stage. For PAP surgery, posterior decompression and the application of pedicle or lateral mass screws were performed without rod assembly during the first surgery stage. Morselized lamina bone autografts were laid lateral to the screws along the facet joints. The second surgery stage involved ACDF followed by the subsequent application of rods immediately after on the same day on the posterior side, ensuring adequate lordosis and good alignment. For both AP and PAP sequences, the interval between the first and second surgery stages was 7 days [7,14]. Extubation was performed in the operating room immediately after the anterior surgery for both surgical sequences. The monitoring of the potential airway compromised secondary to PSTS was observed by obtaining routine sitting lateral cervical radiographs from POD 1 to 5 with patients in full inspiration and neutral forward gaze. The X-ray equipment cassette to tube length was made uniform at 3 feet [9].

### 2.3. Statistical Analysis

A power analysis was performed based on the 31 AP and 26 PAP subjects included for analysis. The results showed that the sample sizes for each group would yield a power of 0.80 to detect an effect size of 0.758 at a two-tailed 0.05 significance level (α). Descriptive measures on the demographic and surgical profiles of the patients are reported. Comparisons between the two means and two proportions were conducted using a *t*-test and *z*-test, respectively. The homogeneity of three or more proportions was assessed using the chi-square test. Differences in the prevertebral soft tissue swelling measurements for each cervical level involved across the different monitoring days were identified using repeated measures analysis of variance. The intra- and interobserver reliability of radiographic values were assessed using the intraclass correlation coefficient (ICC). All statistical analyses were conducted using STATA^®^ V12.0 (StataCorp LLC, College Station, TX, USA) software at a significance level of 0.05. 

## 3. Results

A total of 57 patients was included for the final analysis in the study, including 31 AP surgery and 26 PAP surgery patients. The baseline demographic profiles of the patients in the two surgery groups did not vary with respect to age (*p* = 0.4990), sex (*p* = 0.1820), body mass index (*p* = 0.8480), American Society of Anesthesiologists classification (*p* = 0.5770), diagnosis (*p* = 0.5062), smoking (*p* = 0.4690), or alcohol intake (*p* = 0.3187) (Table 1).

The surgical profiles of the patients in the two types of surgeries did not vary except in terms of total fluid intake during the first and second surgery stages (*p* = 0.0070) and the total operative time during the first and second surgery stages (*p* = 0.0049) (Table 2).

The mean PSTS amounts among patients who underwent AP and PAP surgery significantly differed from the preoperative to postoperative monitoring days at all cervical levels (*p* = 0.0000) (Table 3). Good to excellent intra- and interobserver reliability for all radiographic measurements was observed.

**Figure 3:**
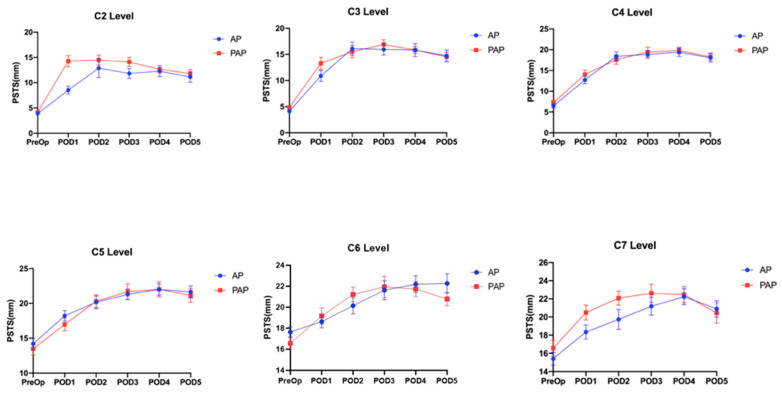
PSTS measurements from the C2 to C7 level between AP and PAP surgery across the different PODs.

The mean PSTS values were significantly higher in PAP (14.27 ± 5.71 mm) than in AP (8.53 ± 4.30 mm) surgery (*p* = 0.0001) at level C2 on POD 1. Among all other levels and across all postoperative monitoring days, however, the mean PSTS amounts between PAP and AP surgery were not significantly different. Additionally, for both groups, we noted that PSTS peaked at PODs 2–4 and, at the same time, that the differences in PSTS with respect to the preoperative measurements were more remarkable at levels C2–4, compared with the lower cervical levels (Figure 3). None of the patients underwent reintubation postoperatively after anterior surgery. Only one patient in the AP group experienced dysphagia at the latest follow-up evaluation of 1 year and 6 months.

## 4. Discussion

Factors to consider when deciding to perform combined anterior–posterior surgery for complex cervical spine cases, especially in degenerative and deformity cases, include the extent of compression, levels of congenital canal stenosis, patient symptoms, spinal alignment, compressive pathology, and the experience and surgical preference of the surgeon [4,15]. Most of the literature, however, only describes single-stage combined anterior–posterior procedures [1,2,13]. At our institution, the AP surgery sequence was initially performed for all combined anterior–posterior procedures, although, in the interim, the PAP surgery sequence was adapted for posterior decompression in the initial stage of surgery, as it was deemed to allow for safer decompression of the compressed anterior part of the cord in the second stage of surgery. Additionally, performing posterior surgery first made the dissection of the posterior soft tissue envelope easier. This slightly flexed posture of the neck lengthened the posterior cervical muscles, thereby making its depth shallower, and thus, performing the anterior surgery after the posterior surgery made positioning the neck in that manner more convenient. PAP surgery also was favorable if an all-pedicle screw construct was preferred, especially in patients with osteoporosis, severe deformity, and large enough pedicles (≥3.5 mm) [6,16,17].

Persistent dysphagia in one patient was probably due to excess anterior and posterior segmental lordotic correction, which could cause stretching of the esophagus coupled with compression of the posterior pharyngeal wall by the vertebra [2,18]. Airway compromise and dysphagia are the most reported complications after anterior cervical surgery, which could be attributed to PSTS [18,19,20,21,22,23,24,25,26]. Combined anterior–posterior cervical spine surgery in particular has been shown to pose a greater risk of complications related to PSTS [27,28,29]. Numerous studies on PSTS have been carried out, but none has explored its relationship with the sequence of surgery in staged multilevel combined anterior–posterior complex cervical spine surgeries [9,16,18,20,25,27]. In this study, the demographic profiles of patients who underwent AP and PAP surgeries were comparable. In terms of surgical profiles, the surgical parameters were similar, except for the total fluid intake and the total operative time during the first and second surgeries. PAP took a longer time to perform as expected: the second stage of PAP involved performing two different approaches (anterior and posterior) on the same day. The total fluid intake during the first and second stages was also greater in PAP than AP, as expected, since PAP involved a longer total operative time. These two factors have been documented in the literature, and researchers have postulated their effects on the development of PSTS [6,7,12,22,27,28]. Accordingly, we suspect that these two may account for the greater PSTS in PAP than in AP on POD1 at the C2 level.

The PSTS amounts differed from the preoperative to postoperative monitoring days at all cervical levels in both PAP and AP surgical groups. Similar to the findings of Suk et al., the swelling was more remarkable at PODs 2–4, and notably, PSTS was greater at levels C2–4 [9]. This was consistent with the observation of Andrew and Sidhu, who indicated that the lower cervical spine has more constrained anatomy that makes it less susceptible to swelling and that there is greater potential retropharyngeal space in the upper cervical spine levels than the lower cervical spine levels [30]. Additionally, we recorded significantly greater PSTS on POD1 at level C2 in PAP surgery, compared with AP surgery. We suspect that this may be due to the contribution of the dependent position of the patient while prone after the anterior surgery during the second surgery stage [8,30,31]. Moreover, since the patient had already undergone anesthesia and intubation before the anterior surgery, this could have caused residual inflammation in the airway that could accrue the PSTS after the anterior surgery in the second stage. Laryngeal edema has been described as an inherent complication of endotracheal intubation, which is not apparent until extubation [31]. Terao et al. reported in their series that seven out of ten patients who underwent combined anterior–posterior cervical spine surgery required postoperative emergency airway management, three of which had postoperative emergency reintubation, and four had prophylactic-delayed extubation all secondary to pharyngeal swelling. They concluded that the incidence of emergency airway management was greater in combined anterior–posterior cervical spine surgery [27]. Wewel et al. reported that, out of seventy-two patients who underwent PAP and AP surgeries, six had delayed extubation, and three had reintubation [32]. The reintubation of 9 patients out of 880 that underwent elective cervical surgery, 3 of whom underwent combined anterior–posterior surgery, was also reported by Schroeder et al. [33]. In all of these accounts, the combined anterior–posterior surgery procedure was performed in a single stage. We have shown that staging combined anterior–posterior surgeries does not result in emergency airway management for both PAP and AP surgeries. However, potential problems should be anticipated in the early postoperative period up to POD4, especially in PAP surgery, when electing to do early extubation due to imminent PSTS.

Pulmonary complications that occur after surgery include atelectasis, pneumonia, bronchitis, bronchospasm, acute respiratory failure, and pulmonary thrombosis. The incidence rates vary from 20 to 69% for atelectasis and 9 to 40% for pneumonia. The total incidence rate varies widely at 10–80% [7]. The occurrence of postoperative pulmonary complications is closely related to the extension of hospitalization and an increase in mortality. Therefore, continuous research has been conducted on the risk factors that can predict the occurrence of pulmonary complications before surgery and on various methods to prevent them. According to the results of previous studies, risk factors that can increase the incidence of postoperative pulmonary complications include sex, age, obesity, smoking history, respiratory disease, underlying diseases other than respiratory diseases, accompanying malignant tumors, anesthesia duration of 4 h or more, and surgical sites [34,35,36,37]. Of these, reducing the duration of anesthesia has proven to be one way to reduce postoperative lung complications in our medical center. Based on this, the period between the two surgeries was decided to be 7 days in consideration of respiratory complications, outpatient schedules, and surgery schedules of surgeons.

The study was limited for being retrospective and could have been made more homogenous if the surgical procedures in both groups were made uniform. In the single-center evaluation, only 57 patients were included. A nonrandom assignment of patients to each surgical group was performed at the discretion of the primary surgeon. It can still be concluded, however, that PSTS in this group of patients was significantly related to the sequence of surgery and that it was greater in PAP than in AP surgery in the immediate postoperative period in the upper airway.

## Figures and Tables

**Table 1:** Demographic profiles of staged AP and PAP patients.

	PAP (*n* = 26)	AP (*n* = 31)	*p*-Value
Age (Year)	64.88 ± 10.45 *	62.77 ± 12.58 *	0.4990
Sex			
Male: Female	14:12 (53.85%:46.15%)	22:9 (70.97%:29.03%)	0.1820
BMI			
<18.5	1 (3.85%)	1 (3.23%)	0.8480
18.5–24.9	12 (46.15%)	17 (54.84%)	
25–29.9	12 (46.15%)	11 (35.48%)	
≥30	1 (3.85%)	2 (6.45%)	
ASA			
1	5 (19.23%)	3 (9.68%)	0.5770
2	14 (53.85%)	18 (58.06%)	
3	7 (26.92%)	10 (32.26%)	
Diagnosis			
Cervical spondylomyeloradiculopathy	22 (84.62%)	24 (77.42%)	0.5062
Secondary Cervical myeloradiculopathy (OPLL, Kyphosis, and Calcium Pyrophosphate Deposition Disease)	2 (7.69%)	4 (12.90%)	
Multilevel herniated cervical disc with spondyloradiculopathy and kyphosis	1 (3.85%)	3 (9.68%)	
Multilevel herniated cervical disc with myeloradiculopathy and kyphosis	1 (3.85%)	0 (0.00%)	
Smoking			
Smoker: Non-smoker	9:17 (34.62%:65.38%)	8:23 (25.81%:74.19%)	0.4690
Alcohol			
Alcoholic: Non-alcoholic	8:18 (30.77%:69.23%)	6:25 (19.35%:80.65%)	0.3187

* Expressed as mean ± standard deviation. The other parameters were given either as *n* (%) or proportion (%). Level of significance set at *p* < 0.05. BMI—Body Mass Index. ASA—American Society of Anesthesiologists Classification. PAP—Posterior–Anterior–Posterior surgical sequence. AP—Anterior–Posterior surgical sequence.

**Table 2:** Surgical profiles of staged AP and PAP patients.

	PAP	AP	*p*-Value
Total Fluid Intake during the first and second stage surgeries (cc)	3238.46 ± 674.73	2732.26 ± 683.07	0.0070
Total Blood Transfusion during the first and second stage surgeries (cc)	0.00 ± 0.00	19.39 ± 60.89	0.1107
Total Urine Output during the first and second stage surgeries (cc)	828.65 ± 308.81	663.39 ± 374.93	0.0783
Total Blood Loss during the first and second stage surgeries (cc)	507.69 ± 293.04	514.52 ± 319.95	0.9339
Total Operative Time during the first and second stage surgeries (min.)	383.58 ± 126.58	307.74 ± 63.00	0.0049
Aggregate Drain Removal Time for both first and second stage surgeries (postoperative day)	6.52 ± 5.78	6.43 ± 5.82	0.8435
Total Drain Output for both first and second stage surgeries (cc)	683.80 ± 232.07	634.12 ± 272.67	0.4810

All parameters were expressed as mean ± standard deviation. Level of significance set at *p* < 0.05. PAP—Posterior–Anterior–Posterior surgical sequence. AP—Anterior–Posterior surgical sequence.

**Table 3:** PSTS measurements at each cervical level between AP and PAP across the different PODs.

Sequence	Baseline	POD1	POD2	POD3	POD4	POD5	*p*-Value ^∞^
C2 Level							
AP	3.90 ± 1.63	8.53 ± 4.30	12.87 ± 7.03	11.82 ± 5.40	12.27 ± 5.98	11.13 ± 5.84	0.0000
PAP	4.18 ± 1.78	14.27 ± 5.71	14.47 ± 4.92	14.11 ± 4.62	12.65 ± 3.72	11.80 ± 4.09	0.0000
*p*-value ^¶^	0.5326	0.0001	0.3317	0.0950	0.7796	0.6222	
C3 Level							
AP	4.13 ± 1.82	10.88 ± 5.77	16.09 ± 7.00	15.94 ± 5.95	15.82 ± 6.92	14.73 ± 6.23	0.0000
PAP	4.85 ± 1.93	13.27 ± 5.98	15.52 ± 5.92	16.90 ± 4.74	15.84 ± 3.42	14.52 ± 4.51	0.0000
*p*-value ^¶^	0.1519	0.1309	0.7404	0.5118	0.9861	0.8905	
C4 Level							
AP	6.52 ± 3.10	12.70 ± 5.02	18.41 ± 5.83	18.84 ± 5.11	19.38 ± 5.52	18.05 ± 5.72	0.0000
PAP	7.29 ± 2.69	14.03 ± 5.18	17.62 ± 5.89	19.43 ± 5.93	19.80 ± 3.87	18.29 ± 4.60	0.0000
*p*-value ^¶^	0.3241	0.3281	0.6105	0.6890	0.7416	0.8598	
C5 Level							
AP	14.17 ± 4.51	18.22 ± 4.20	20.16 ± 5.13	21.29 ± 4.23	21.99 ± 4.55	21.65 ± 4.89	0.0000
PAP	13.47 ± 4.49	16.97 ± 4.65	20.3 ± 4.74	21.67 ± 5.70	22.03 ± 5.46	21.13 ± 4.89	0.0000
*p*-value ^¶^	0.5601	0.2912	0.9185	0.7770	0.9800	0.6901	
C6 Level							
AP	17.64 ± 2.69	18.63 ± 3.31	20.15 ± 4.39	21.62 ± 5.00	22.20 ± 4.44	22.27 ± 5.11	0.0000
PAP	16.55 ± 2.84	19.17 ± 3.96	21.22 ± 3.55	21.95 ± 5.23	21.74 ± 3.70	20.79 ± 3.29	0.0000
*p*-value ^¶^	0.1412	0.5818	0.3225	0.8085	0.6774	0.2090	
C7 Level							
AP	15.41 ± 3.77	18.34 ± 4.37	19.73 ± 6.10	21.18 ± 5.48	22.24 ± 4.92	20.90 ± 5.09	0.0000
PAP	16.58 ± 4.29	20.49 ± 4.17	22.08 ± 3.94	22.62 ± 5.12	22.47 ± 4.53	20.43 ± 5.74	0.0000
*p*-value ^¶^	0.2801	0.0639	0.0965	0.3147	0.8601	0.7466	

PSTS—Prevertebral Soft Tissue Swelling measured in millimeters. PODs—Postoperative days. PAP—Posterior–Anterior–Posterior surgical sequence. AP—Anterior–Posterior surgical sequence. The measurements are given as mean ± standard deviations. Level of significance set at *p* < 0.05. ^¶^—According to the independent *t*-test. ^∞^—According to the repeated measures analysis of variance.

## Data Availability

Not applicable.
